# Transcriptional Control of the Multi-Drug Transporter ABCB1 by Transcription Factor Sp3 in Different Human Tissues

**DOI:** 10.1371/journal.pone.0048189

**Published:** 2012-10-25

**Authors:** Radka Gromnicova, Ignacio Romero, David Male

**Affiliations:** Department of Life, Health and Chemical Sciences, The Open University, Milton Keynes, Bucks, United Kingdom; Indiana University School of Medicine, United States of America

## Abstract

The ATP-binding cassette (ABC) transporter ABCB1, encoded by the multidrug resistance gene MDR1, is expressed on brain microvascular endothelium and several types of epithelium, but not on endothelia outside the CNS. It is an essential component of the blood-brain barrier. The aim of this study was to identify cell-specific controls on the transcription of MDR1 in human brain endothelium. Reporter assays identified a region of 500 bp around the transcription start site that was optimally active in brain endothelium. Chromatin immunoprecipitation identified Sp3 and TFIID associated with this region and EMSA (electrophoretic mobility shift assays) confirmed that Sp3 binds preferentially to an Sp-target site (GC-box) on the MDR1 promoter in brain endothelium. This result contrasts with findings in other cell types and with the colon carcinoma line Caco-2, in which Sp1 preferentially associates with the MDR1 promoter. Differences in MDR1 transcriptional control between brain endothelium and Caco-2 could not be explained by the relative abundance of Sp1:Sp3 nor by the ratio of Sp3 variants, because activating variants of Sp3 were present in both cell types. However differential binding of other transcription factors was also detected in two additional upstream regions of the MDR1 promoter. Identification of cell-specific controls on the transcription of MDR1 indicates that it may be possible to modulate multi-drug resistance on tumours, while leaving the blood brain barrier intact.

## Introduction

Microvascular endothelium in the brain is a key component of the blood-brain barrier, which controls the movement of nutrients into the CNS and excludes many toxic molecules from the CNS. Brain endothelial cells are connected by continuous tight junctions that confer low permeability to ions and hydrophilic molecules [1]. They also express several members of the ATP-binding cassette (ABC) super family, of which the most important is ABCB1, encoded by the multi-drug resistance gene, MDR1 [2], [3]. (ABCB1 is often referred to as p-gp1.) Additional multi-drug resistance proteins (MRP) are located at the blood-brain barrier, including MRP-1, -2 and -4 (ABCC1, 2, 4) as well as breast-cancer resistance protein, (ABCG2) [4]. These features contribute to blood-brain barrier function and are responsible for maintaining brain homeostasis and normal neuronal activity. However, the multi-drug transporters also prevent the entry of many potentially useful drugs into the CNS.

The multi-drug transporters are expressed in complex tissue-specific patterns. For example ABCB1 is expressed on brain endothelium, but not on endothelia that lack barrier properties. However, it is present on a variety of other cell-types, including intestinal epithelium and on cells from the proximal kidney tubules. Moreover ABCB1 is induced by a wide variety of drugs, and is present on many tumours, rendering these cell types resistant to treatment with some cytotoxic drugs. Hence, ABCB1 is subject to a variety of transcriptional controls, which affect both constitutive expression and induction in different cell types [5]. In order to selectively modulate ABCB1 expression in different cell types, it is essential to understand these cell-specific controls. The aim of this study was to identify transcription factors that control ABCB1 expression in human brain endothelium.

**Figure 1:**
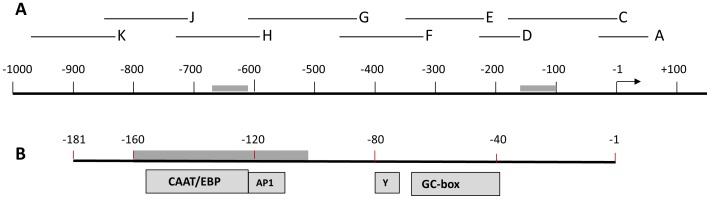
Diagram of the human MDR1 proximal promoter. (A) The promoter of *MDR1* from −1000 bp to +100 bp is illustrated diagrammatically. Numbering is based on the proposed transcription-start site at +1 (arrow). Two regions of high inter-species homology are indicated by grey bars. The positions of nine overlapping probes (A,C,D,E,F,G,H,J,K) used in EMSA are indicated above the main scale. (B) The segment corresponding to probe C is expanded below, to show potential target sites of transcription-factors in epithelial cells and tumours, as well as a Y-box and a GC-rich box. CAAT/EBP = CAAT enhancer binding protein; AP1 = Activating protein-1.

There have been many studies on the expression of ABCB1 in epithelial cells and tumours which have identified transcription factor binding sites in the proximal promoter of MDR1 (Fig. 1). The MDR1 promoter lacks a TATA-box, but does have an inverted CCAAT sequence (Y-box). Consequently, it has been proposed that transcription is initiated by NF-Y binding to the Y-box, rather than TFIID, which binds to TATA-boxes [6]. Furthermore, it has been shown that CCAAT-enhancer-binding protein-β (CEBP/β) and Specificity-protein-1 (Sp1), promote assembly of the RNA-polymerase II complex to the Y-box [7]. However, it is essential to note that these studies are based on tumours and epithelial cells. In contrast, there is little information on transcription control of MDR1 in brain endothelium. One study on rat brain endothelium has identified a distal promoter element ∼10 kb upstream of the transcription start site, which responds to steroids [8], but there is no data on the proximal promoter, which is expected to contain the key transcription factor target sites for gene expression. In this study we have analysed the proximal part of the MDR1 promoter up to 1 kb upstream from the transcription start site (Fig. 1).

Previous work from our laboratory has compared transcription factor profiles in endothelium from the brain and other tissues, with the aim of identifying distinctive sets of transcription factors which could induce or maintain the phenotype of barrier endothelium [9]. These studies identified a role for the Sp family, YY1 and TFIID in the regulation of the human transferrin receptor (TFR) promoter, leading to the proposal that Sp3 is required for TFR expression in brain endothelium. Subsequently, it was found that Sp3 is also involved in the control of occludin, a component of tight junctions [10]. This led us to the hypothesis that Sp3 could be part of a more general system, controlling many proteins which are co-expressed in brain endothelium, but not other endothelia.

Four members of the Sp-family have been identified in brain endothelium, of which Sp3 and Sp1 are most abundant. Brain endothelium expresses particularly high levels of Sp3 in comparison with lung and dermal endothelium [9]. Sp1 is the prototype of a large family of transcription factors which bind GC-rich segments [11], [12]. Sp1 itself is thought to be a constitutive factor that enhances the transcriptional initiation of numerous genes whereas Sp3 has been reported to act as an activator or repressor depending on the cell type and conditions [13].

In view of our previous results on transcriptional control of TFR and occludin, this study focuses on the role of the Sp-family and other transcription factors, which have differential expression in brain and non-brain endothelium. Underlying this study is the objective of maintaining ABCB1 expression at the blood brain barrier, while modulating expression on tumours or other epithelial barriers. Hence the results with brain endothelium were compared with results from the well-studied epithelial colon carcinoma line, Caco-2. We also investigated the possible role of other transcription factors (CEBPβ, NF-Yα, PCAF, YB1 ) that have been reported to control MDR1 expression in epithelial cells or tumours.

## Results

### Expression of ABCB1 (p-gp1) in Endothelial and Epithelial Cell Lines

**Figure 2:**
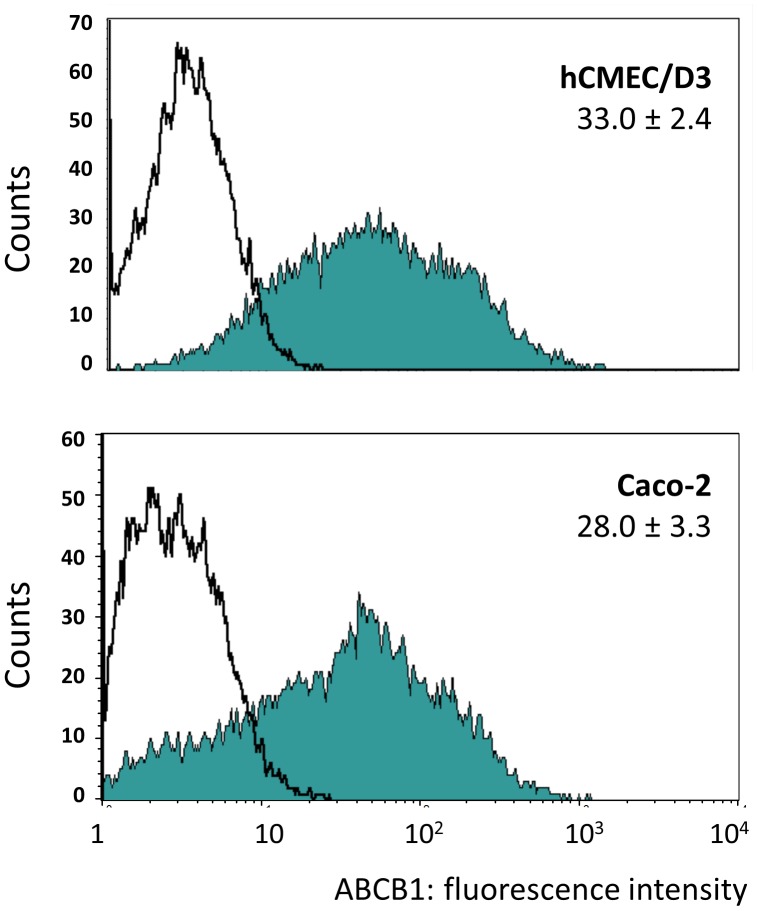
Expression of ABCB1 on hCMEC/D3 and Caco-2. Representative FACS plots of ABCB1 expression on hCMEC/D3 and Caco-2 cells. The filled histograms are from immunofluorescence staining with a primary antibody to ABCB1 (MRK16) and open histograms are with an isotype-matched negative control as primary antibody. The figures show the mean (± SEM) of the median fluorescence of four independent experiments with each cell-type. (The secondary antibody, the staining procedure and FACS acquisition parameters were identical in all experiments.).

The expression of ABCB1 was compared in the human cerebral microvascular endothelial cell line/D3 (hCMEC/D3) and the epithelial tumour line Caco-2, by FACS (Fig. 2). Both cell lines express ABCB1 over a wide range with similar median fluorescence. In order to investigate how ABCB1 expression is controlled in brain endothelium and to compare different cell types, promoter reporter vectors were generated containing segments of the proximal MDR1 promoter region (Fig. 1). This region was chosen because it contains a previously reported transcription start site active in epithelial cells (+1), and the segment up to position +43 was required for full promoter activity [5]. The target sites of several transcription factors that are reported to be required for gene expression in epithelial cells or tumour cells are shown expanded on the region between −181 and −1 bp (Fig. 1b).

**Figure 3:**
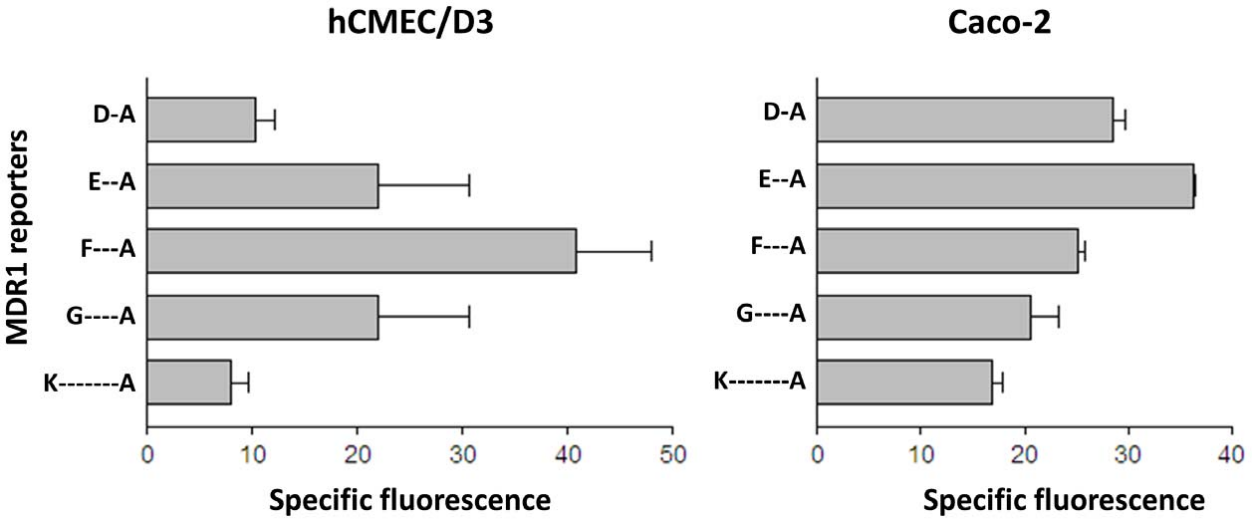
Activity of MDR1 promoter gene segments. Specific activity of five MDR1 promoter-reporter vectors was measured in hCMEC/D3 and Caco-2 cells by FACS. The promoter segments correspond to the regions shown in figure 1. D-A = −228 to +43: E-A = −338 to +43: F-A = −458 to +43: G-A = −617 to +43: K-A = −986 to +43. The data shows mean ± SEM of the median fluorescence, minus background from 3 independent determinations.

To identify active regions of the MDR1 promoter, five promoter-reporter vectors were transfected into hCMEC/D3 or Caco-2 cells and transcriptional activity determined by GFP expression (Fig. 3). The aim of the experiments was to detect whether the profile of activity of the reporter vectors was different in the two cell types, indicating that different sets of transcription factors were active on MDR1 in the two cells. All of the vectors showed some promoter activity compared to the empty reporter vector and analysis of variance (ANOVAR) on the data from each cell indicated that the vectors showed a pattern of differential activity (p<0.05 in both cases). The region −457 to +43 was clearly most active in hCMEC/D3 cells, whereas the segment −338 to +43 was most active in Caco-2 cells. The results indicated that different elements of the MDR1 promoter were required for optimal gene expression in the two cell types.

For comparison, the vectors were also transfected into primary lung or myometrial microvascular endothelium. In these experiments the vectors E-A and F-A showed a low level of activity, and the other vectors were inactive (data not shown). However, the transfection efficiency in these experiments was low (5–10%) as determined using the CMV-promoter positive control vector. Therefore we cannot be certain that the low level of expression in non-brain endothelium is due to lack of appropriate transcription factors for MDR1. It was not possible to carry out a comparison with primary brain endothelium due to the very limited amount of live human brain tissue available.

### Transcription Factor Binding to the MDR1 Promoter

**Figure 4:**
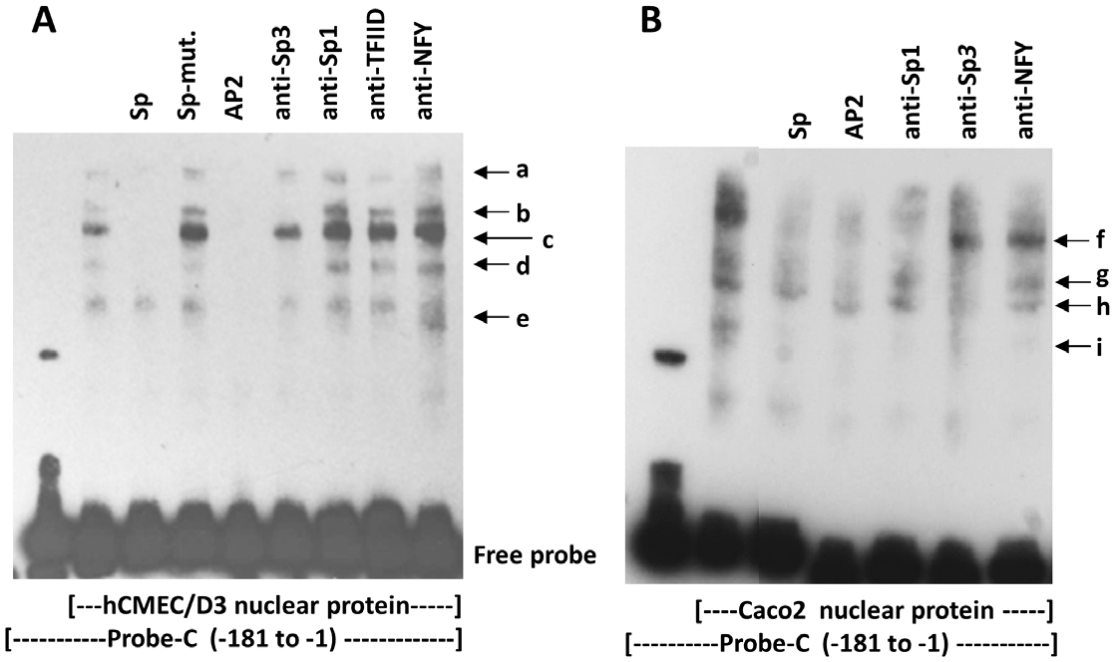
Interaction of Sp transcription factors with the MDR1 promoter. (A) EMSA with probe-C in lanes 1–9, and hCMEC/D3 nuclear protein in lanes 2–9. Protein-binding was blocked with a consensus Sp oligonucleotide, a mutated Sp oligonucleotide (Sp-mut.) and a consensus AP2 oligonuceotide (lanes 3–5). Supershifts were attempted with antibodies to Sp3, Sp1, TFIID and NFY (lanes 6–9). The nuclear protein produced five shifted bands, of which three (b,c,d) were reduced by anti-Sp3. (B) EMSA with probe-C in lanes 1–7 and Caco-2 nuclear protein in lanes 2–7. The nuclear proteins produced 4 major shifted bands (f–i). Protein-binding was blocked with consensus oligonucleotides for Sp or AP2 (lanes 3 & 4). Supershifts with specific antibodies (lanes 5–7) indicated that band ‘f’ was produced by Sp1, bands ‘g’ and ‘h’ by Sp3. Band ‘i’ was substantially reduced by all treatments.

To determine whether nuclear proteins from hCMEC/D3 cells bind to the MDR1 promoter, EMSA was carried out with a series of overlapping probes (Fig. 1a) to screen for transcription factor binding. The screen showed strong binding of nuclear proteins to probes C, E, F and G, with weaker bands associated with probes H and K (screening data is not shown, but see Fig. 4. and below). Hence, the data imply that the major protein interactions were with target sites in the regions −457 to −229 and −161 to −40. These regions lie within segment F-A [−457 to +42] that was most active in the reporter assays.

Our earlier studies indicated that the transcription factors Sp3 and YY1 were critically important in controlling expression of TFR and occludin in brain endothelium. We therefore proposed that Sp3 could be more generally used by brain endothelium to control tissue-specific gene expression. Two Sp-factor binding sites are located in the GC-rich box of MDR1 (Fig. 1b), however studies in epithelial cells and tumours indicated that it is Sp1 that targets this site [5], [6].

To investigate interaction of transcription factors with this region, EMSA was carried out using probe-C [−181 to −1] and nuclear protein from hCMEC/D3 or Caco-2 (Fig. 4). The blots show 5 bands (a-e) with hCMEC/D3 nuclear proteins, and 4 bands (f-i) with Caco-2 nuclear protein. The transcription factors binding to region C were further analysed by blocking the EMSA with unlabelled oligonucleotides or supershifting with antibodies to transcription factors (Fig. 4).

In brain endothelium, the results show that bands ‘a-d’ were blocked by a consensus Sp oligonucleotide with sequence homology to the GC-box. This indicates that bands ‘a-d’ are due to proteins binding to the GC-box. Furthermore, all of the bands were blocked by an AP2-consensus oligonucleotide which has two homology regions in probe-C, one corresponds with the GC-rich box, and the other lies at position [−64 to −89], between the AP1 site and the Y-box (Fig. 1). This pattern of blocking indicates that bands ‘a-d’ are due to proteins binding to the GC-rich box, and ‘e’ is binding to a region immediately upstream of the Y-box. Moreover, there was no blocking with consensus oligonucleotides corresponding to CEBP, NF1 and AP1 (data not shown), which confirms that the transcription factors were binding to the GC-box and not to the CEBP target, the Y-box or the AP1 target region, respectively. Antibody to Sp3 removed bands ‘b’ and ‘d’ and substantially reduced the strongest band ’c’, indicating that these bands are due to Sp3 binding. Antibodies to Sp1, TFIID and NF-Y had no effect (Fig. 4).

In Caco-2 cells, band ‘f’ was blocked by an Sp consensus oligonucleotide and the AP2 consensus oligonucleotide. This band was supershifted by antibody to Sp1, implying that Sp1 is a component of this band. Bands ‘g’ and ‘h’ were partly blocked by Sp and AP2 oligonucleotides and removed by antibody to Sp3, indicating that Sp3 targeting the Sp consensus sequence, is a component of these complexes. Band ‘i’ was removed by all treatments (both oligonucleotides and antibodies), which suggests a weak non-specific interaction with probe-C.

**Figure 5:**
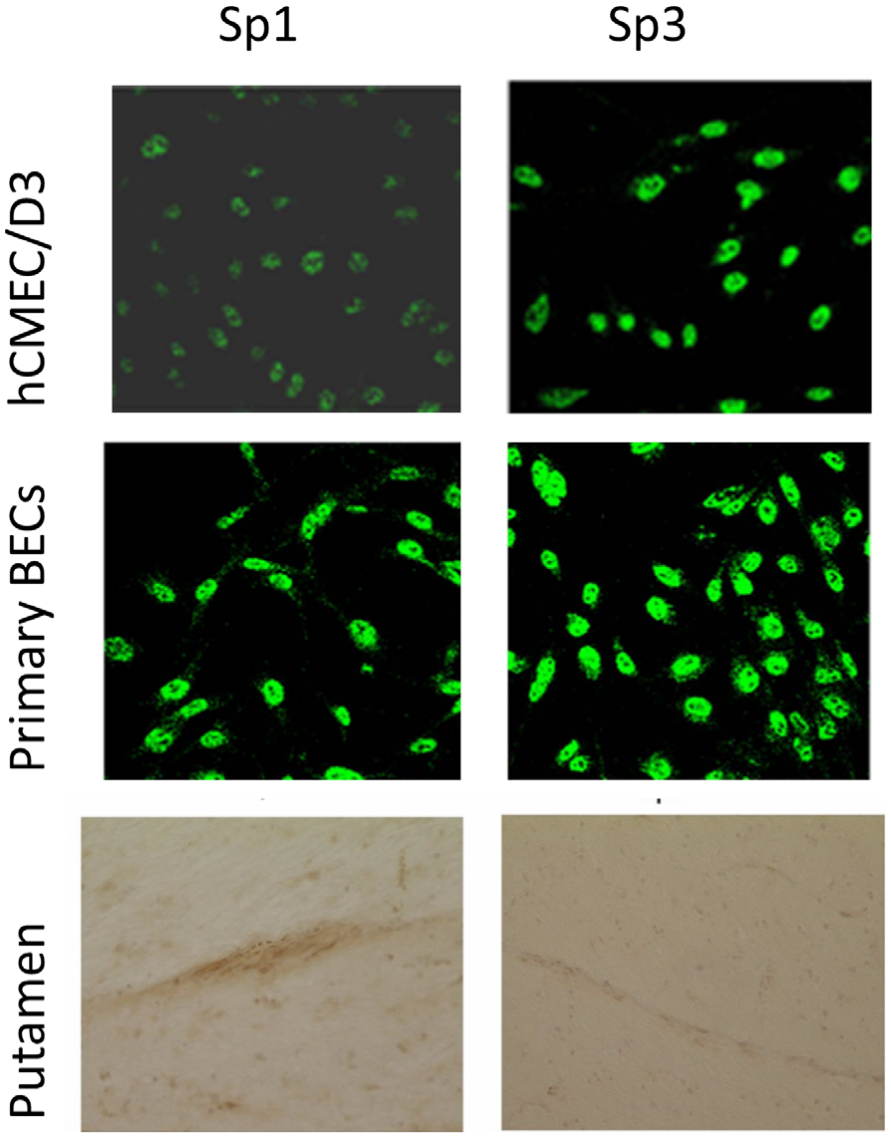
Expression of Sp1 and Sp3 in human brain endothelium in vitro and in situ. Expression of Sp1 and Sp3 by immunofluorescence in hCMEC/D3 cells and primary human brain endothelium and by immunohistochemistry in blood vessels in the putamen of human brain.

These results implied that Sp3 associated with the GC-rich box in brain endothelium whereas Sp1 was associated with the promoter in Caco-2 cells. This result is in agreement with previous results on epithelial cells and tumours. We therefore investigated the relative expression and isoforms of Sp3 and Sp1 in brain endothelium. Immunofluorescence of hCMEC/D3 cells, primary brain endothelium and brain endothelium in situ, all show that Sp1 and Sp3 are both present in the endothelial line as well as primary brain endothelial cells (Fig. 5). Previously we have found that both Sp1 and Sp3 can be detected in hCMEC/D3 by EMSA-supershift assay with a consensus Sp oligonucleotide probe. Moreover, the activity of Sp3 is higher in brain endothelium, than non-brain endothelium [9]. The different techniques, all indicate that Sp3 is strongly expressed in brain endothelium, although Sp1 is also present.

**Figure 6:**
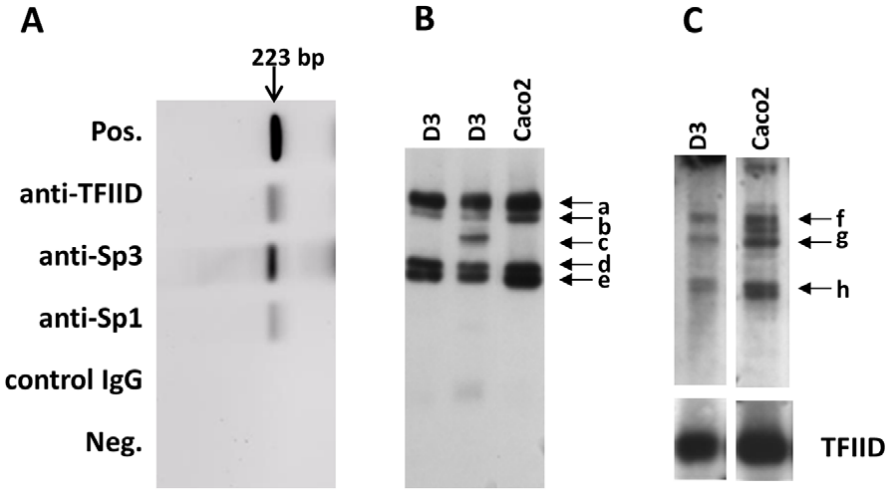
Trancription factor binding to the MDR1 promoter in brain endothelium. (A) ChIP was performed on hCMEC/D3 cells with antibodies to TFIID, Sp3, Sp1 and a control IgG. The proximal MDR1 promoter was detected by PCR, with primers spanning the region −223 to −1 (see figure 1). Full length promoter was used as a positive control (Pos.) No chromatin was used as an additional negative control (Neg.). (B) Western blotting of nuclear proteins from hCMEC/D3,(D3) or Caco-2 cells, with antibody to Sp3. Bands ‘a’ and ‘b’ correspond to the activating/suppressive variants of Sp3. Bands ‘d’ and ‘e’ correspond to non-activating variants. (C) Western blotting of nuclear proteins from hCMEC/D3 (D3), or Caco-2 cells, with antibody to Sp1. Previously reported variants of Sp1 correspond to bands ‘f’ and ‘g’. The blot was stripped and reprobed with antibody to TFIID as a positive control for loading and transfer.

To further investigate the relative importance of Sp1 and Sp3, chromatin immunoprecipitation (ChIP) was carried out using antibodies to a number of transcription factors and sheared chromatin from resting hCMEC/D3 cells. The precipitated DNA was detected by PCR using primers spanning the region D+C (see Fig. 1). The results show that Sp3 is strongly associated with the proximal promoter in hCMEC/D3 cells but Sp1 was only weakly associated (Fig. 6a). Interestingly, TFIID associates with the MDR1promoter in brain endothelium, even though it does not contain a TATA box. ChIP with antibodies to Pit-1, c-myb, GATA-2, YY-1 and Sp2 were all negative (data not shown).

It was notable that the upper Sp3 band seen in EMSA with brain endothelium (Fig. 4, band ‘b’), was absent from the Caco-2 cells. We therefore investigated the possibility that Caco-2 cells lacked an Sp3 isoform, which could explain the lack of Sp3 binding from these cells. Sp3 is known to be produced in four principle variants, two of which (∼117 kDa) may be activating or suppressive depending on the gene and cell type whereas the two smaller bands (∼96 kDa) are not activating [14]. To test this hypothesis, nuclear proteins from the two cell types were examined by Western blotting (Fig. 6b). The results show that both cells express all four variants of Sp3 (bands a,b,d,e). In addition some preparations of hCMEC/D3 cells expressed an additional band (‘c’) corresponding to a sumoylated form of the lower molecular weight variants [14]. Note that while it is valid to assess the relative amounts of the different isoforms of Sp1 or Sp3 in a single lane by this method, one cannot compare the amounts of Sp1 and Sp3, since the effectiveness of the anti-Sp1 and anti-Sp3 antibodies was different in Western blotting.

Since two Sp3 bands appeared in EMSA with hCMEC/D3 nuclear protein and probe-C (Fig. 4), we hypothesised that these two bands corresponded to the two high molecular weight forms of Sp3, seen on the Western blots (Fig. 6b). To test this theory, we devised a novel two dimensional gel system with EMSA in the first dimension and SDS-PAGE in the second dimension. The resulting two dimensional gel was blotted and stained with anti-Sp3 in a Western blot. The results indicated that the two high molecular weight forms of Sp3 bound to the MDR1 promoter (probe-C) whereas the low molecular weight forms did not bind (data not shown). This result was true for Sp3 (in nuclear protein) obtained from either hCMEC/D3 or Caco-2.

Two previously reported isoforms of Sp1 (Fig. 6c, bands ‘f’ and ‘g’, 106 kDa and 95 kDa) were detected in both hCMEC/D3 and Caco2 cells. There was an additional smaller band (‘h’) in all of the cells. Low molecular-weight proteins reacting with anti-Sp1 antibodies have been reported previously, but the relationship to full-length Sp1 has not been defined. It is known that Sp1 activity is controlled by proteolytic degradation, and it is therefore possible that band ‘h’ represents a degradation product caused by loss of the N-terminal activation domain. These data show that the selective binding of Sp3 to the MDR1 promoter in brain endothelium is not related to the presence of activating or non-activating isoforms of Sp3, or the absence of Sp1 isoforms in brain endothelium.

The data suggested that activating variants of Sp3 bind to the MDR1 promoter in brain endothelium. Therefore, we would have liked to test whether removal of Sp3 results in a secondary reduction in ABCB1 expression. However, we have previously attempted to knockdown Sp3 with siRNA in hCMEC/D3 cells, and found that it resulted in cell death; this is not surprising in view of the large number of genes that have GC-rich boxes in their promoter and which require Sp-factors for their activation. We therefore tried an alternative method, using mithramycin which interferes with the binding of transcription factors to GC rich regions and might therefore be expected to reduce ABCB1 expression [15]. hCMEC/D3 cells were cultured in 200 nM mithramycin for 3 days and then analysed for ABCB1 expression by immunofluorescence and FACS. Contrary to the hypothesis, mithramycin-treated cells had a significantly higher level of ABCB1 expression, than untreated cells (Mithramycin-treated, 30.1±1.88 : Control, 18.8±0.97, Mean of median fluorescence, n = 3, p<0.01, by Student’s t-test). This result may be explained by the fact that mithramycin is itself a substrate of ABCB1, and cells that express low levels of ABCB1 are selectively inhibited in their growth/survival compared with high expressers, an effect that has been previously shown with puromycin, another cytostatic ABCB1 substrate [16].

**Figure 7:**
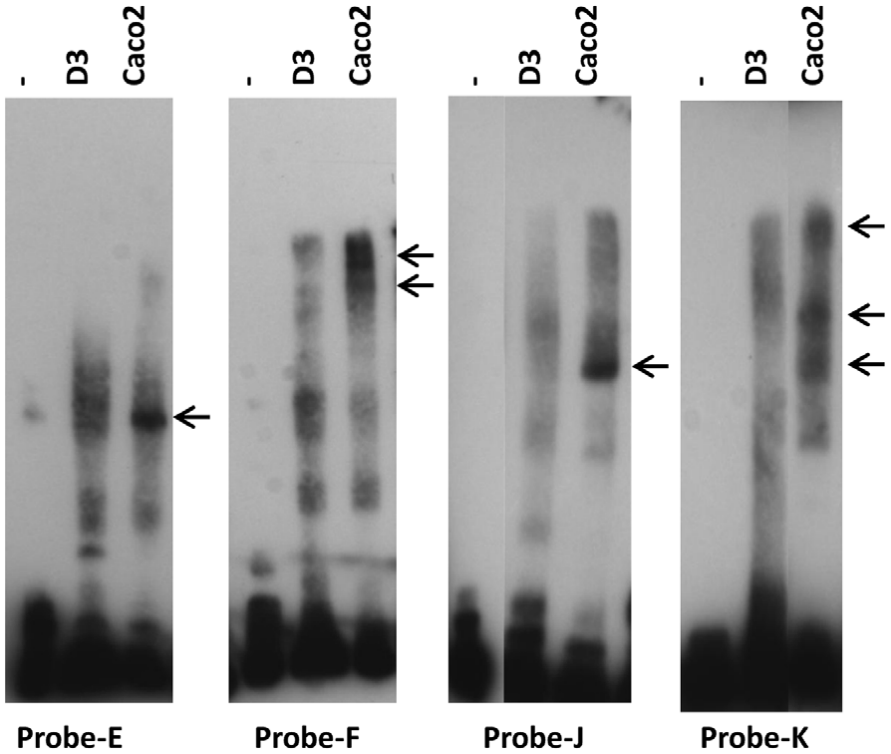
Differential binding of transcription factors to the MDR1 promoter in brain endothelium and Caco-2. EMSA with probes E, F, J and K and nuclear proteins from hCMEC/D3 (D3) compared with Caco2. Arrows indicate bands that are strongly expressed in the Caco-2 cells, but absent or at low levels in hCMEC/D3 cells.

Since the differential binding of Sp3 to the GC-box is not explained simply by its incidence or variants within the cells, we compared the binding of transcription factors from the two cell types to upstream regions of the promoter by EMSA. The results showed differential protein binding to probes-E, -F, -J and -K (Fig. 7). Regions E and F, which bind hCMEC/D3 transcription factors, are part of the active promoter in these cells (Fig. 3). Although there was only a low level of hCMEC/D3 nuclear-protein binding to probes J and K, there was one strong band from Caco-2 which bound to probe-J and three bands associated with probe-K. Taken together the data suggest that Sp3 binds preferentially to the MDR1 promoter in brain endothelial cells, whereas Sp1 is more active in Caco-2 cells. However there is also differential transcription factor binding in the region −457 to −229 in hCMEC/D3 cells and at −986 to −711 in Caco-2 cells, which may explain the different profiles of promoter activity seen in the two cell types.

## Discussion

The data presented in this paper demonstrate that the transcription factor Sp3 associates with the GC-rich box in the proximal MDR1 promoter in brain endothelium. In comparison, published work on other cell types has shown that it is Sp1 that drives MDR1 transcription [5]. Our experiments confirm the previous findings that Sp1 binds the MDR1 GC-rich box in Caco-2 cells, but indicate that MDR1 is controlled differently in brain endothelium. Our previous work has shown that Sp3 is also required to control expression of occludin and TFR in brain endothelium [9], [10]. Collectively, the studies suggest that a number of genes, characteristic of the blood-brain barrier phenotype use Sp3 for their transcriptional activation.

The Western blots of Sp3 confirm previous findings of two long and two short forms of Sp3. The two long forms of Sp3 both contain two transcription-activation domains but the two short forms lack the second (3′) activation domain. Production of the variants is complex, but in essence the two short forms are produced from transcript variants that lack exon-3, encoding the second activation domain [14], [17]. Interestingly, the 2-dimensional gels indicated that only the long forms of Sp3 bound to the MDR1 promoter, even though all variants contain the DNA-binding zinc-fingers. This suggests that other transcription factors interact with the long forms of Sp3 to promote DNA-binding. We have examined a number of cell types that either express or do not express ABCB1 (lung microvascular endothelium, MDR1-negative: astrocytes, low expression : the kidney epithelial cell line ciPTEC [18], MDR1-positive); all of the cells have the high molecular weight forms of Sp3. Therefore the expression of MDR1 cannot be explained solely on the basis of selective expression of the high molecular weight, active forms of Sp3.

The MDR1 gene lacks a TATA-box, but does have an invert CCAAT-box. It has therefore been assumed that assembly of RNA-polymerase would involve NF-Y (which binds to CCAAT) rather than TFIID (which binds to TATA) [7]. However it was notable that anti-NF-Y antibodies did not supershift the nuclear-protein complex binding to probe-C. It has also been proposed that CCAAT-enhancer binding protein-β (CEBP/β), bound to its target (Fig. 1) would enhance the binding of NF-Y [5]. However, anti-CEBP/β antibody did not produce a supershift in the EMSA with probe-C, nor did a CEBP-oligonucleotide block the nuclear-protein complexes. Moreover, the oligonucleotide-blocking assays with the Sp consensus sequences showed that the major nuclear protein complex was bound to the GC-box with a minor element upstream of the CCAAT-box. In short, there was absolutely no evidence for transcription factor binding to the CCAAT-box or a function for CEBP/β in human brain endothelial cells. In contrast, TFIID was detected in association with the promoter in ChIP (Fig. 6a). Since there is no target site for TFIID in that segment, it suggests that it is not directly bound to the promoter but is associated with it, by interaction with another transcription factor. In this context, it has been shown that the second activation domain of Sp3 (encoded by exon-3) can bind to TFIID, and recruit it to TATA-less promoters [11].

The question arises of why Sp3 should bind to the MDR1 promoter, rather than Sp1? It has been proposed that Sp3 will compete efficiently with Sp1 on promoters that contain multiple Sp target sites clustered within the proximal promoter [19]. It is notable therefore that the MDR1 promoter contains two closely linked Sp target sites and hence is a candidate gene for control by Sp3. How it is controlled in different cells will depend on the variants of Sp3 that are expressed, and on other transcription factors that interact with Sp3.

Interestingly, we sometimes noted an Sp3 band in the hCMEC/D3 cells (Fig. 6b, band ‘c’) corresponding to the sumoylated form of the shorter variants; there was no evidence for the sumoylated form of the long isoforms (127 kDa). This indicates a possible mechanism for increasing MDR1 transcription in these cells, ie destruction of the inhibitory form of the transcription factor Sp3. The complex pattern of Sp3 variants seen in different cell types may help to explain, how Sp3 can act as an activator on some genes/cells, but a repressor on others.

The data indicate that additional tissue-specific controls affect MDR1 transcription, ie Sp3 is necessary but not sufficient for MDR1 expression in brain endothelium whereas Sp1 is preferentially used in Caco-2 cells. Additional transcriptional controls may lie in regions E and F (hCMEC/D3) and in regions E, J and K (Caco-2) since there is differential binding of transcription factors to these segments (Fig. 7). However we were unable to identify the transcription factors using the panel of supershift antibodies listed in table 1, which includes all the transcription factors previously identified as acting on MDR1 in other cell types.

**Table 1:** Primary antibodies used in ChIP, EMSA, Western blotting and immunofluorescence.

Specificity	Species^a^	Source	Clone/Serum	Use^b^
Sp 1 (PEP 2)	Rabbit	Santa Cruz Bio.	Sc-59	ChiP WB IF IHC
Sp 1 (IC6)	Mouse	Santa Cruz Bio.	Sc-420	EMSA
Sp 3 (D-20)	Rabbit	Santa Cruz Bio.	Sc-644	ChIP WB IF EMSA IHC
TFIID (TBP) (SI-1)	Rabbit	Santa Cruz Bio.	Sc-273	ChIP WB
TFIID (TBP) (N-12)	Rabbit	Santa Cruz Bio.	Sc-204	EMSA
NFY-A	Rabbit	Novus Biol.	NBP1-67884	EMSA
YB1	Rabbit	Novus Biol.	NBP1-40492	EMSA
CEBP β	Rabbit	Novus Biol.	NB100-91681	EMSA
PCAF	Rabbit	Sdix	3459.00.02	EMSA
NFY	Rabbit	AbD Serotec	AHP298	EMSA
Pit-1	Goat	Santa Cruz Bio.	Sc-16288	ChIP
GATA-2 (CG2-96)	Mouse	Santa Cruz Bio.	Sc-267	ChIP EMSA
c-Myb	Rabbit	Santa Cruz Bio	Sc-7874	ChIP EMSA
P-gp1	Mouse	Kamiya Biomed.	MRK-16	IF FACS

a Rabbit and goat antibodies are polyclonal. Mouse antibodies are monoclonal. All antibodies were affinity purified.

b Antibodies were used for Chromatin immunoprecipitation (ChIP), Western blotting (WB, [200–500 ng/ml]), Immunofluorescence (IF, [10–20 µg/ml]), electrophoretic mobility shift assays (EMSA, [20–100 µg/ml]) or immunohistochemistry (IHC) (10 µg/ml).

In previous studies, we have shown that the presence or absence of YY1 was correlated with the on/off state of occludin and the transferrin receptor in endothelial cells from different tissues [10]. We could not however detect YY1 bound to the region of the MDR1 promoter analysed here – ie up to 1 k base upstream of the transcription start. The absence of YY1-binding indicates that it is not required for the expression of MDR1 in brain endothelium, although it could still have a role in preventing transcription in non-brain endothelia.

It is possible that there are additional sites controlling MDR1 expression that are more distant from the transcription start-site, and it has previously been shown that a site 10 k bases upstream is responsive to steroids, via activation of the pregnane-X receptor [8], although expression of this receptor is reported to be very low on hCMEC/D3 cells [20]. However, transcriptional activators usually bind within 1–2 kb of the transcription start site, and the reporter data presented here (Fig. 3) indicates that this is also true for MDR1 in brain endothelium.

In summary, the data supports the proposal that Sp3 is required for activation of MDR1 in brain endothelium, whereas the cell types examined previously use Sp1 to activate MDR1. Sp3 may recruit TFIID to the MDR1 promoter by binding to the second activation domain of Sp3, although TFIID does not itself bind directly to the gene. Additional transcription factors acting upstream of the GC-box in the region E-F (Fig. 1) are differentially active in brain endothelium and are required for optimal transcription in these cells.

The identification of cell-type specific controls on ABCB1 expression is important, because it indicates that it may be possible to maintain ABCB1 expression on brain endothelium, while reducing it on other cell types [21], for example in tumour therapy. Conversely, if one wanted to reduce ABCB1 expression on brain endothelium, it could be done without removing its protective function from other cell types.

## Materials and Methods

### Cell Cultures

The human brain microvascular endothelial cell line, hCMEC3/D3 [22], was grown on collagen-coated plates in EGM-2 MV medium supplemented with 2.5% foetal bovine serum, hydrocortisone, VEGF, epidermal growth factor (EGF), insulin-like growth factor I (IGF-I), human fibroblast growth factor (FGF), ascorbic acid, gentamicin sulphate and amphotericin-B. hCMEC/D3 cells were used at passage 21–30. Primary human brain microvessel endothelium was obtained from surgical resection, undertaken to treat epilepsy, with the informed consent of the patient. The cells were isolated from a small area of unaffected tissue at the tip of the temporal lobe, by collagenase/dispase digestion and isolation on BSA and percoll gradients as previously described [23].

The human colon-derived epithelial cell line Caco-2 [24] was maintained in monolayer cultures in MEM with 10% foetal bovine serum, penicillin and streptomycin.

### Flow Cytometry, Immunofluorescence and Immunohistochemistry

hCMEC/D3 and Caco-2 cells were analysed for expression of ABCB1 by flow cytometry. Cells were washed in HBSS without Ca^++^/Mg^++^, detached from the flasks with trypsin/EDTA, and fixed with 2% paraformaldehyde in PBS for 30 m. The cells were washed, resuspended in 5 g/l BSA in PBS and 2×10^5^ cells were stained with mouse IgG2a antibody to ABCB1 (p-gp1, Clone MRK16, Kamiya Biomedical) at a final concentration of 15 µg/ml in 50 µl for 2 h. The secondary antibody was 1/100 rabbit anti-mouse-FITC (Sigma) for 1 h. Cells were washed twice with PBS between the antibody incubations and before FACS analysis.

For detection of transcription factors in vitro by immunofluorescence, endothelial cells were grown to confluence on collagen coated coverslips before fixation with 4% paraformaldehyde and staining, with 20 µg/ml antibody to Sp1 or Sp3, followed by 1/100 FITC-labelled anti-mouse-IgG as previously described [9]. Immunohistochemistry for Sp3 and Sp1 was carried out on cryostat sections of the unaffected putamen from an individual with multiple sclerosis [25] using primary antibodies to Sp1 and Sp3 (see table 1) at 10 µg/ml. In all immunofluorescence and immunocytochemistry experiments, isotype-matched primary antibodies at equivalent dilution to the test antibody were used for the negative controls.

### Promoter-vectors, Transfection and FACS Analysis

The MDR1 promoter segments were prepared by PCR, using primers corresponding to positions in the MDR1 promoter indicated in Table 2 and as a template, a vector containing the full MDR1 promoter region, kindly supplied by Professor Mike Waring (University of Cambridge). The amplified segments were cloned into pGlowTOPO (Invitrogen) and the sequences of the gene segments and their correct orientation, upstream of the GFP-reporter gene was checked by PCR before use in transfection assays. pGLOW containing a strong CMV-promoter was used as positive control and to check transfection efficiency. Empty, closed vector was used as a negative control to determine background fluorescence.

**Table 2:** Sequences of probes in MDR1 promoter region.

Probe	Position in NT007933.15*	Size (bp)
A	25263083−25263002	82
C	25263224−25263044	181
D	25263272−25263204	69
E	25263382−25263252	131
F	25263500−25253361	140
G	25263664−25263478	187
H	25263775−25263645	131
J	25263887−25263755	133
K	25264029−25263866	164

* *Homo sapiens*, chromosome-7 genomic contig. Reference sequence.

**Table 3:** Consensus dsDNA blocking oligonucleotides.

Target	Sequence of consensus oligonucleotide
Sp	5′-ATTCGATCGGGGCGGGGCGAGC-3′
Sp mutant	5′-ATTCGATCGGTTCGGGGCGAGC-3′
AP1	5′-CGCTTGATGAGTCAGCCGGAA-3′
AP2	5′-GATCGAACTGACCGCCCGCGGCCCGT-3′
c/EBP	5′-TGCAGATTGCGCAATCTGCA-3′
TFIID	5′-GCAGAGCATATAAAATGAGGTAGGA-3′
YY1	5′-CGCTCCCCGGCCATCTTGGCGGCTGGT-3′
c-Myb	5′-TACAGGCATAACGGTTCCGTAGTGA-3′
GATA	5′-CACTTGATAACAGAAAGTGATAACTCT-3′

hCMEC/D3 cells were plated at 2–6×10^5^ cells per well on 6 well plates in 2 ml of EGM2-MV medium without antibiotics but supplemented with serum and growth factors and cultured until 60% confluent. For each transfection, 2 µg of DNA was diluted in 250 µl of OptiMEM® medium and 10 µl of Lipofectamine™ 2000 was diluted in 250 µl OptiMEM® Medium. DNA-Lipofectamine™ 2000 complexes were produced according to the manufacturer’s protocol (Invitrogen) and 500 µl was added directly to each well and incubated at 37°C for 12 h. The complexes were then removed from the wells and EGM2-MV medium without antibiotics but with reduced serum (1%) and growth factors was added and cells cultured for a further 72 h before analysis of reporter GFP expression. In these conditions transfection efficiency of hCMEC/D3 cells was 40–60% as distinguished by increased size/granularity [10]. Caco-2 cells were transfected under similar conditions, for 6 hours before removal of the complexes, and were then maintained in MEM with 10% FCS, for 54 h before assay (transfection efficiency 30–45%). Transfected cell monolayers were washed in HBSS, detached with 0.25% Trypsin-EDTA, centrifuged at 300 g for 5 min and resuspended in HBSS without phenol-red and were then analysed immediately by FACS. In the FACS analysis, the transfected cells were gated by their forward-scatter and side-scatter, and the median fluorescence of 10,000 transfected cells was determined ie. non-transfected cells were not included in the analysis. Specific fluorescence is defined as the median fluorescence of cells transfected with a reporter vector minus the median fluorescence of cells transfected with the empty vector. Results are shown as the mean ±SEM of the specific fluorescence from 3 independent determinations.

### Chromatin Immunoprecipitation (ChIP) Assays

Chromatin was prepared as described previously [10] and fragmented using an enzymatic shearing cocktail to generate DNA fragments of 1–2 kb in length. Two large flasks, containing ∼9×10^6^ resting hCMEC/D3 cells were used for each chromatin preparation to produce 2 ml of sheared chromatin, sufficient for eight immunoprecipitations.

The immunoprecipitation stages were based on a kit supplied by Upstate. Each 250 µl sheared chromatin lysate was pre-cleared with 40 µl of salmon sperm DNA (GibcoBRL)/Protein-A Agarose-50% slurry for 1 h at 4°C. The cleared sample was then incubated overnight at 4°C with 2 µg antibody specific for Sp1, Sp3, TFIID, YY1, Pit-1, c-myb or GATA-2 (Santa Cruz, Biotechnology). 60 µl of salmon sperm DNA/Protein-A Agarose slurry was then added and further incubated for 1 h at 4°C with rotation. The protein-A agarose/antibody/chromatin complex was pelleted and washed successively with low-salt buffer, high-salt buffer, LiCl complex wash buffer and Tris/EDTA. The bound chromatin was then eluted by two 15 min incubations in 250 µl 1% SDS, 0.1 M NaHCO_3_ and the two eluates were pooled.

The protein-DNA cross-links were reversed, by adding 20 µl of 5 M NaCl to each eluate and heating at 65°C for 4 h. Then, 10 µl of 0.5 M EDTA, 20 µl 1 M Tris-HCl, pH 6.5 and 2 µl of 10 mg/ml Proteinase K was added and incubated for 1 h at 45°C. After addition of 3 µl glycogen (2 µg/µl), DNA was recovered by phenol/chloroform extraction and ethanol precipitation and redissolved in Tris/EDTA, pH 8.4 for use in PCR analyses. PCR for ChIP assays used primers spanning the segment DC (see Fig. 1), annealing at 50°C for 30 s, with an extension time of 60 s at 74°C and 35 cycles of amplification. The amplified segments were analysed using 2% agarose gels in TAE buffer.

### Electrophoretic Mobility Shift Assays (EMSAs)

Nuclear protein extracts were isolated from cells as previously described, diluted to 2–5 mg/ml and stored at −80°C before use [10]. Biotinylated EMSA probes, corresponding to MDR1 promoter fragments (Table 2, Fig. 1a) of <200 bp, were prepared by PCR from the full length MDR1 promoter template using one conventional oligonucleotide primer and one biotinylated primer. The PCR products were run on 2% agarose gels and fragments migrating at the appropriate size were excised from the gel, extracted and purified (SpinPrep Gel DNA kit, Novagen). Probes were diluted to 1–2 ng/µl for use in the assay.

The EMSA was based on a kit from Pierce (LightShift Chemiluminescent EMSA). Briefly, 1–2 ng of biotinylated EMSA probe was incubated for 20 min with 10 µg of nuclear protein extracts in a final volume of 20 µl binding buffer which included MgCl_2_, NP40, and poly dIdC at the manufacturer’s recommended concentrations. In some cases the nuclear proteins were pre-incubated for 20 min with 0.4 µg oligonucleotides (cold blocking assay) or with 1–3 µg antibody against specific transcription factors (supershift assay). Details of the blocking oligonucleotides are given in Table 3 and supershift antibodies are described in Table 2.

The electrophoretic mobility shift assays were then conducted on 6% polyacrylamide gels in 0.5× TBE. The gel was prerun at 80 V for 60 min. The samples were then loaded and electrophoresed at 90 V for 70 min, at which time the free probes were near the bottom of the gel. The complexes were transferred by wet blotting (in 0.5× TBE) onto nylon membrane (Amersham Hybond N+) at 100 V for 1 h. The nucleic acids were immobilised on the blots by UV-crosslinking (120 mJ/cm^2^) and the position of the biotinylated probes identified by incubation with streptavidin-horseradish peroxidase, and chemiluminescent detection according to the manufacturer’s protocol.

### Western Blotting

Nuclear protein samples were prepared in non-reducing or reducing conditions and separated in 6% SDS-polyacrylamide gels. The proteins were transferred onto nitrocellulose membranes which were blocked with 5% non-fat milk in PBS containing 0.05% Tween-20 at 4°C overnight. Immunoblotting was performed using anti-Sp3 or anti-Sp1 (200 ng/ml) (Santa Cruz Biotechnology) as primary antibodies, for 1 h at room temperature. Membranes were then washed five times in PBS containing 0.05% Tween-20 and incubated with secondary antibody goat anti-rabbit IgG conjugated to HRP (1∶ 5000) (Invitrogen) for 1 h at room temperature. Immunoblots were washed five times in PBS/Tween-20 and once in PBS only. The enhanced chemiluminescence detection system (Amersham) was used for visualization.
